# Untangling statistical and biological models to understand network inference: the need for a genomics network ontology

**DOI:** 10.3389/fgene.2014.00299

**Published:** 2014-08-29

**Authors:** Frank Emmert-Streib, Matthias Dehmer, Benjamin Haibe-Kains

**Affiliations:** ^1^Computational Biology and Machine Learning Laboratory, Faculty of Medicine, Health and Life Sciences, Center for Cancer Research and Cell Biology, School of Medicine, Dentistry and Biomedical Sciences, Queen's University BelfastBelfast, UK; ^2^Institute for Bioinformatics and Translational Research, UMITHall in Tyrol, Austria; ^3^Bioinformatics and Computational Genomics Laboratory, Princess Margaret Cancer Centre, University Health NetworkToronto, ON, Canada

**Keywords:** gene regulatory networks, computational genomics, statistical inference, mathematical modeling, systems biology, epistemology, genomics network ontology

## Abstract

In this paper, we shed light on approaches that are currently used to infer networks from gene expression data with respect to their biological meaning. As we will show, the biological interpretation of these networks depends on the chosen theoretical perspective. For this reason, we distinguish a *statistical perspective* from a *mathematical modeling perspective* and elaborate their differences and implications. Our results indicate the imperative need for a *genomic network ontology* in order to avoid increasing confusion about the biological interpretation of inferred networks, which can be even enhanced by approaches that integrate multiple data sets, respectively, data types.

## 1. Introduction

The post-genomic era possesses considerable challenges for the development of novel data analysis approaches. A reason for this necessity stems from the fact that in order to conduct a sensible data analysis, a method and the data from a unit that needs to fit optimally together, in order to extract the maximal amount of robust information from the data set. However, high-throughput technologies used in genomics generate data with novel characteristics for which, usually, no off-the-shelf methods are available. This is especially true for approaches that aim to infer large-scale networks from gene expression data (Friedman, 2004; Wille et al., 2004; Zhang et al., 2012).

The purpose of our manuscript is to untangle two distinct but related perspectives that are currently, in our opinion, inadvertently mixed-up in the literature. Specifically, in this paper, we focus exclusively on methods for “obtaining” networks from gene expression data and two opposing concepts from which methods are derived. We call the first concept category the *statistical perspective* (Stats P) and the second the *mathematical modeling perspective* (Math MP). In the following, we, first, define what we mean by a statistical and mathematical modeling perspective, second, we compare them with each other to show that they have complementary purposes and meanings and, third, we provide an explanation of possible sources for this confusion. Finally, we suggest a potential solution to avoid future antilogies by establishing a *genomics network ontology*.

Overall, the goal of this paper is to provide conceptual clarity in the interdisciplinary area of network inference from high-throughput data, because with the anticipated availability of novel high-throughput technologies, e.g., on the single-gene level or from imaging technologies, the inference of regulatory networks from the integration of such data types will become increasingly important. Hence, this problem could dramatically accelerate if not tackled in its early phase.

## 2. Statistical perspective

The first perspective we describe in this paper is the *statistical perspective*. By this we mean any approach that applies a statistical inference method to a gene expression data set in order to draw conclusions about the biochemical interactions between genes and gene products without requiring further constraints or assumptions, e.g., regarding the underlying biological mechanisms. The result of such an approach can be used to make predictions about the interactions of genes and gene products for constructing a network representation of the biochemical interactions. For reasons of clarity, we provide an explicit definition for the resulting network.

**Definition 1**. *A network that has been inferred from gene expression data by the application of a statistical inference method is called a “gene regulatory network.” In this network, nodes correspond to genes or gene products and edges correspond to biochemical interactions of any type. The network can be directed or undirected*.

For this definition, we used the term gene regulatory network (GRN) because it is frequently used (Wang et al., 2006; Hecker et al., 2009). However, for reasons of completeness, we would like to note that in the literature, there are also various alternative notations used instead of GRN (Haibe-Kains and Emmert-Streib, 2014), but for the following discussion the nomenclature is not of relevance.

### 2.1. Purpose of the statistical perspective

From the above definition one can see that a statistical perspective is very general as it does not require biological information of any kind about the underlying mechanisms within a biological cell. Instead, biological information can be gained from the inference process about the biochemical interactions.

Now, the question is what type(s) of biochemical interaction(s) can be identified by statistical network inference methods? The answer to this question is actually only partially given by the data type used for the inference. Since the inference is based on gene expression data, which provide information about the abundance of mRNAs only rather than binding information of, e.g., a transcription factor to a promoter region or protein-protein binding, gene regulatory networks defined in the above sense provide information about *general regulatory interactions* between regulators and their potential targets; gene-gene interactions, and potential protein-protein interactions (e.g., in a complex) (de Matos Simoes et al., 2013). In other words, if a network inference method predicts the interaction between two genes or gene products then there is no systematic way to find out which particular biochemical interaction type this is, due to the nature of gene expression data. Hence, this is not a shortcoming of any network inference method, but the data themselves.

In brief, the purpose of the statistical perspective is to make predictions regarding the presence of interactions and directions of interactions within gene regulatory networks. However, such a method or its components are not required to have a meaningful biological interpretation (in contrast, see Section 3.1) in the sense that these emulate, e.g., a biological process.

### 2.2. Methods for inferring gene regulatory networks

There are many examples where such networks have been studied (Margolin et al., 2006; Werhli et al., 2006; Meyer et al., 2008; Stolovitzky et al., 2009; Emmert-Streib et al., 2012); see Table 1 for a brief overview of some widely used methods. All of these methods have in common that they estimate *statistical independence relations* for random variables, by different inference approaches, to construct a GRN component-wise.

**Table 1:** **A brief overview of statistical network inference methods that have been introduced in recent years (first column) and the key methods (second column) on which the inference algorithms are based on to estimate interactions**.

**Name**	**Method**	**References**
Aracne	Mutual information, DPI	Margolin et al., 2006
C3Net	Maximal mutual information	Altay and Emmert-Streib, 2010
BC3Net	Bagging C3Net	de Matos Simoes and Emmert-Streib, 2012
ENNET	Gradient boosting	Slawek and Arodz, 2013
GENIE3	Regression	Huynh-Thu et al., 2010
GGM	Full partial correlation	Wille et al., 2004
MRNet	Conditional mutual information	Meyer et al., 2008
NIMEFI	Ensemble feature importance methods	Ruyssinck et al., 2014

### 2.3. Who uses the statistical perspective

The statistical perspective is the preferred approach in computational biology, computational genomics, biostatistics and bioinformatics, where the goal is to extract (infer) information from a given data set to predict population aspects of its.

## 3. Mathematical modeling perspective

In a molecular biological context, which is our focus, the *mathematical modeling perspective* aims to provide a realistic model for the transcription of DNA into RNA and the translation of RNA into proteins. However, there are also simpler models that focus on the former part only. In general, the detail level of such models varies considerably (see Section 3.2).

### 3.1. Purpose of the modeling perspective

The purpose of the mathematical modeling perspective is to increase our understanding of dynamical properties of a system and to derive behavioral features thereof that can then be used for making predictions about a natural system. This is possible because, usually, such a system is meant to form a mechanistic model of a natural (biological) system or process. Seminal models in this context are for instance the model of a neuron (Hodgkin and Huxley, 1952), a heart (NOBLE, 1960) or enzyme kinetics (Schnell and Mendoza, 1997). Simply put, the purpose of a mathematical model is to emulate a natural system as good as possible with respect to its dynamical activity. Additionally, even its components have a meaningful biological interpretation.

### 3.2. Methods to model transcription regulation

Over the last years, many different models have been introduced to model the transcription regulation of genes (Gardner and Faith, 2005; Karlebach and Shamir, 2008; Ribeiro, 2008). These models vary on, e.g., the level of their detail complexity, the closeness to biological reality or the time complexity of the simulations.

Among the first and simplest approaches to models of GRNs are Boolean Networks and Probabilistic Boolean Networks (Kauffman, 1993; Shmulevich et al., 2002); see Table 2. These models assume a discrete activity level of genes, which can be either on (1) or off (0). For this reason such models are called *logical models*. Discrete Boolean Networks and Probabilistic Boolean Networks provide a simplistic representation of the transcriptional activity rather than a detailed biological formulation of molecular processes.

**Table 2:** **A brief overview of some mathematical modeling methods that are used to model the transcription regulation of genes**.

**Type**	**Model**	**References**
Logical model	Boolean network	Kauffman, 1993
Logical model	Probabilistic Boolean network	Shmulevich et al., 2002
Continuous model	Ordinary differential equations	Chen et al., 2004
Continuous model	Michaelis-Menten and Hill kinetics	Van den Bulcke et al., 2006
Single molecule	Gillespie's stochastic simulation algorithm	Gillespie, 1976
Single molecule	Approximate SSA	Ribeiro et al., 2006

Models allowing to simulate a continuous gene activity dynamics are, e.g., systems of coupled ordinary differential equations (ODEs) or the Gillespie's stochastic simulation algorithm (SSA). Different models in this category can be distinguished based on the biological details that are modeled. The two major model categories focus either on transcription regulation or transcription and translation events. Hence, the latter model type is closest with respect to the phenomenologically observable biological mechanisms. Another difference between these models is if the average activity of a population of cells is simulated or the activity of a single cell (Ribeiro et al., 2006; Kandhavelu et al., 2012).

### 3.3. Who uses the mathematical modeling perspective

The mathematical modeling perspective is very popular in mathematical biology, theoretical biology, systems biology, biochemistry and biophysics.

## 4. Comparison of the two perspectives

**Figure 1:**
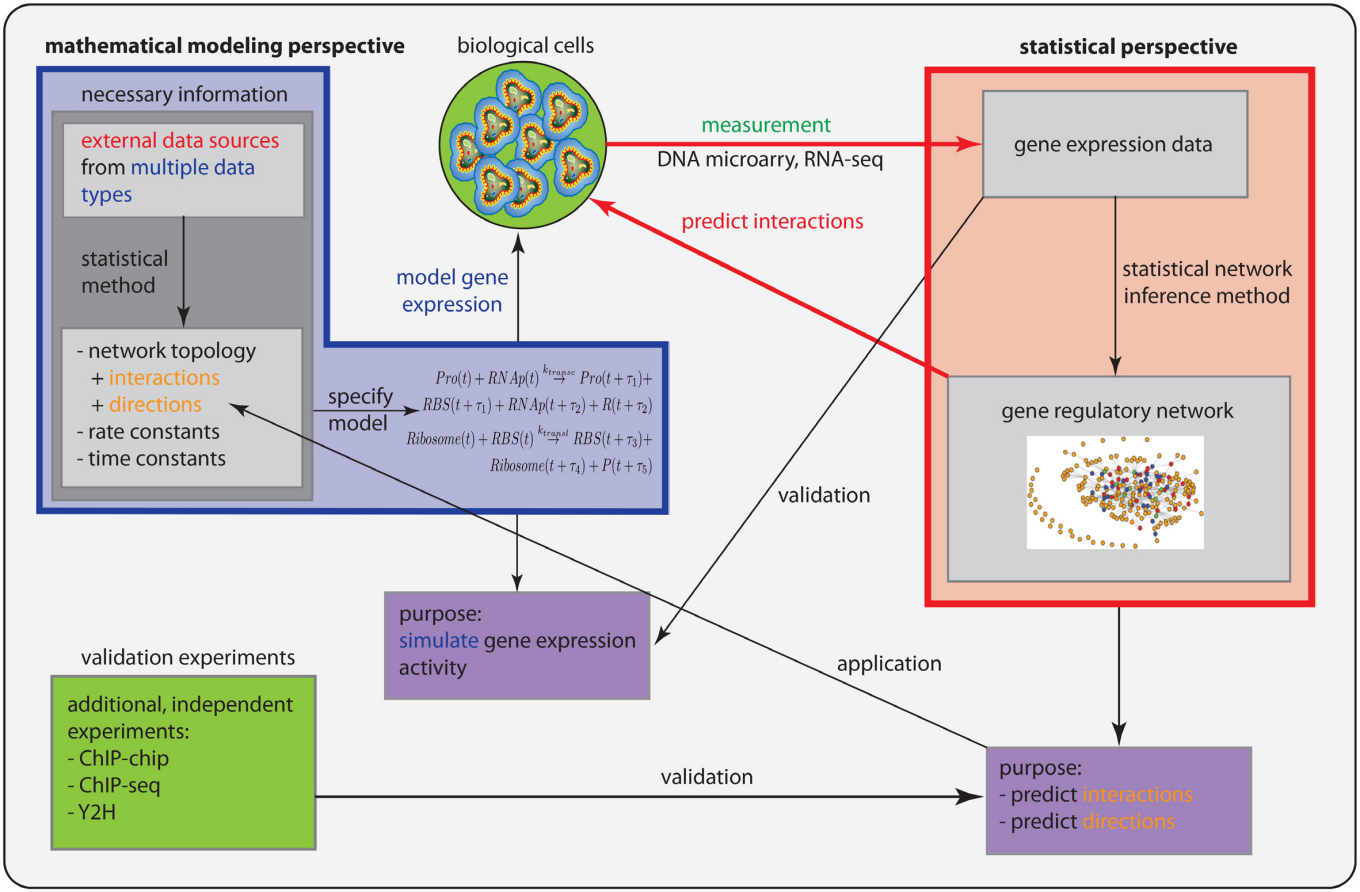
**Schematic comparison of the statistical perspective (red) and the mathematical modeling perspective (blue)**.

In Figure 1, we show a schematic overview and a comparison of the statistical perspective (red box) and the mathematical modeling perspective (blue box), discussed individually in the previous sections.

However, there are three important differences. (1) Prior information: From a statistical perspective, there is no prior information required in addition to a gene expression data set to conduct an analysis. In contrast, in order to specify a mathematical model, information about the connectivity of the genes is needed and also the parameter values for the rate and time constants. (2) Purpose: The purpose of both perspectives is different. Whereas the goal of a statistical approach is to predict interactions between genes and gene products, and potentially their directionality (not all methods try to do this), the purpose for a mathematical model is to simulate gene expression levels. (3) Validation: From the former points, there results an immediate consequence for the validation of the models. In order to validate predicted interactions, additional data from different data types are needed that provide direct information about biochemical binding activity. For instance, information about the protein-DNA binding from ChIP-chip or ChIP-seq experiments can be used to identify interactions between transcription factors and regulated genes. Also, proteomics data, e.g., from Y2H experiments, provide information about protein binding and protein complex formation that can be used in this respect. In contrast, mathematical modeling approaches are validated by gene expression data. In Table 3, we show a summary of these differences.

**Table 3:** **Comparison of properties, features and requirements of statistical and mathematical models that fall under the category statistical perspective (Stat P) and mathematical modeling perspective (Math MP)**.

	**Stats P**	**Math MP**
Necessary prior information	No	Yes
Predict interactions	Yes	No
Predict directions	Yes	No
Simulate expression activity	No	Yes
Validation data: ChIP-chip	Yes	No
Validation data: Y2H	Yes	No
Validation data: gene expression	No	Yes

## 5. The theoretical perspective matters practically

In the following, we make an attempt for explaining the unfortunate amalgamation of the statistical perspective and the mathematical modeling perspective that might form sources for confusions and provide also some examples from the literature.

First, the interdisciplinary character of the problem to infer networks from gene expression data provides such a source by itself. The reason for this is that different subject areas have a different educational focus toward either the statistical perspective (computational biology—see Section 2.3), or the mathematical modeling perspective (mathematical biology—see Section 3.3). Hence, there is a natural plurality of complementary perspectives in this field that can get entangled easily.

Second, some early papers in this field presented in review articles methods from a statistical perspective and the mathematical modeling perspective side-by-side, which might have given the misleading impression that there is actually no difference between both (D'haeseleer et al., 2000; Gardner and Faith, 2005). These influences can be also observed in more recent review papers following the same structure (Bansal and di Bernardo, 2007; Hecker et al., 2009; Chai et al., 2014). Furthermore, there are research papers that contributed to this impression (Hoon et al., 2003). However, we would like to point out that there are also positive examples were this misleading presentation has been avoided, essentially, by focusing on one perspective only (de Jong, 2002; Kim et al., 2003; Markowetz and Spang, 2007; Karlebach and Shamir, 2008; Emmert-Streib et al., 2012).

In our opinion, the very best example to understand the imperative need for a clear distinction of both perspectives is given if one asks for the meaning of networks inferred from gene expression data. Because the answer is: It depends on your perspective. The explanation of this, forms our third example.

Suppose, you assume a mathematical modeling perspective and you use a model that emulates transcription regulation without consideration of a protein level. In this case, one concludes that the inferred network corresponds to a transcription regulation network. On the other hand, if you assume a statistical perspective using any of the algorithms listed in Table 1, the meaning of this network is a mixture of a transcription regulation network and a protein interaction network (Gardner and Faith, 2005; de Matos Simoes et al., 2013); as we will see below.

The crucial question is, how can it happen that we get from the same gene expression data set networks with different (but overlapping) meaning? The reason for this is that not the data alone define the meaning of an inferred network, but the combination of the data and the method. Specifically, the mathematical modeling perspective requires us to define a biological model with a well-defined biological meaning, whereas the statistical perspective does not. Instead, all methods in Table 1 estimate *statistical independence relations* (Emmert-Streib et al., 2012), because this is required to estimate causal relations (Pearl, 2000). Unfortunately, a statistical independence relation cannot be equated with either “transcription regulation” or “protein-protein interaction,” because it is not a model of reality (biology), but a statistical model.

So far, we established that in the former case a network is a transcriptional regulatory network but are still lacking a meaning in the latter case. However, this gap can be naturally filled by thinking one step further addressing the question of *network validation*. Here by network validation we mean that we compare an inferred network with an experimentally determined biological network. By using transcriptional regulatory networks and protein interaction networks it has been shown that a network inferred from a statistical perspective contains a significant number of interactions of both types (de Matos Simoes et al., 2013). Hence, such a network is a mixture of a transcriptional regulatory network and a protein interaction network. This is of course not a surprise but has already been discussed before (Gardner and Faith, 2005).

For reasons of clarity, we add a fourth example, discussing a question that we encountered frequently in various arguments. The question is, how can a network whose interactions are a composition of transcription regulations and protein interactions provide a valid model of the biology system? The answer is, such a network does not aim to be a biological model by itself, but it aims to be a statistical model that allows to make predictions about constituting parts of the biological system (see Table 1 for the purpose of the statistical perspective and the mathematical modeling perspective).

## 6. Subjective objectivity

We waited until this point in our paper to provide an explanation for our chosen terminology, i.e., why we prefer the term “perspective” over, e.g., “formalism” to denote the two conceptual categories—statistical perspective and mathematical modeling perspective—because based on the arguments presented above, this is easy to understand now. A “perspective” is commonly associated with an individual point of view, which can be perceived as *subjective*. On the other hand, a “formalism” appears to be *objective*. In science, one strives for objectivity, but every assumption one makes is subjective by nature. Hence, despite the fact that any *statistical formalism* and any *mathematical modeling formalism* is fully objective, the selection process itself to pick either one of the formalisms is subjective. From this, it appears very natural to chose “perspective” over “formalism” to indicate explicitly that despite of the objectivity of the two formalisms the resulting interpretation depends on the selection too; as we have seen in our discussion above.

## 7. A genomics ontology for inferred networks

Due to the expected increase in the next years with respect to more data, but also novel biotechnologies to generate further high-throughput data types, we are facing an urgent need to organize the vocabulary for inferred networks. Specifically, data integration becomes more and more important implying that from 7 different data types $21\left(=\left(\begin{array}{c} 7\\ 2 \end{array}\right)\right)$ different network types can be inferred using two different data types only, whereas integrating three data types gives already $35\left(=\left(\begin{array}{c} 7\\ 3 \end{array}\right)\right)$ different network types. Considering the fact that the method itself has also an influence on the meaning of such networks it is easy to imagine that there are immense problems waiting for us to be addressed in order to avoid mounting confusion in this area. For this reason, we suggest to establish a *genomics network ontology* that provides a systematic vocabulary for networks inferred from biological, biomedical and clinical omics data.

## 8. Conclusion

The purpose of our paper was to generate awareness for the important distinction between a statistical perspective and a mathematical modeling perspective when inferring networks from gene expression data. However, this is only one of many epistemological problems we are currently facing in genomics (Dougherty, 2008) generated by the intricate interplay between large-scale high-throughput data and mathematical inference procedures. We hope that such problems receive more appreciation in the future because their neglection can lead to disastrous effects, especially when entering translational research (Dougherty, 2009).

### Conflict of interest statement

The authors declare that the research was conducted in the absence of any commercial or financial relationships that could be construed as a potential conflict of interest.
